# NOViSE: a virtual natural orifice transluminal endoscopic surgery simulator

**DOI:** 10.1007/s11548-016-1401-8

**Published:** 2016-06-17

**Authors:** Przemyslaw Korzeniowski, Alastair Barrow, Mikael H. Sodergren, Niels Hald, Fernando Bello

**Affiliations:** 1 Centre for Engagement and Simulation Science, Department of Surgery and Cancer, Imperial College London, Chelsea and Westminster Hospital, 369 Fulham Road, SW10 9NH London, UK; 2 Department of Surgery and Cancer, Imperial College London, London, UK

**Keywords:** Cosserat rod, Flexible endoscope, Natural orifice surgery, NOTES, Virtual reality simulator

## Abstract

**Purpose:**

Natural orifice transluminal endoscopic surgery (NOTES) is a novel technique in minimally invasive surgery whereby a flexible endoscope is inserted via a natural orifice to gain access to the abdominal cavity, leaving no external scars. This innovative use of flexible endoscopy creates many new challenges and is associated with a steep learning curve for clinicians.

**Methods:**

We developed *NOViSE*—the first force-feedback-enabled virtual reality simulator for NOTES training supporting a flexible endoscope. The haptic device is custom-built, and the behaviour of the virtual flexible endoscope is based on an established theoretical framework—the Cosserat theory of elastic rods.

**Results:**

We present the application of *NOViSE* to the simulation of a hybrid trans-gastric cholecystectomy procedure. Preliminary results of face, content and construct validation have previously shown that *NOViSE* delivers the required level of realism for training of endoscopic manipulation skills specific to NOTES.

**Conclusions:**

VR simulation of NOTES procedures can contribute to surgical training and improve the educational experience without putting patients at risk, raising ethical issues or requiring expensive animal or cadaver facilities. In the context of an experimental technique, *NOViSE* could potentially facilitate NOTES development and contribute to its wider use by keeping practitioners up to date with this novel surgical technique. NOViSE is a first prototype, and the initial results indicate that it provides promising foundations for further development.

**Electronic supplementary material:**

The online version of this article (doi:10.1007/s11548-016-1401-8) contains supplementary material, which is available to authorized users.

## Introduction

### NOTES

**Fig. 1:**
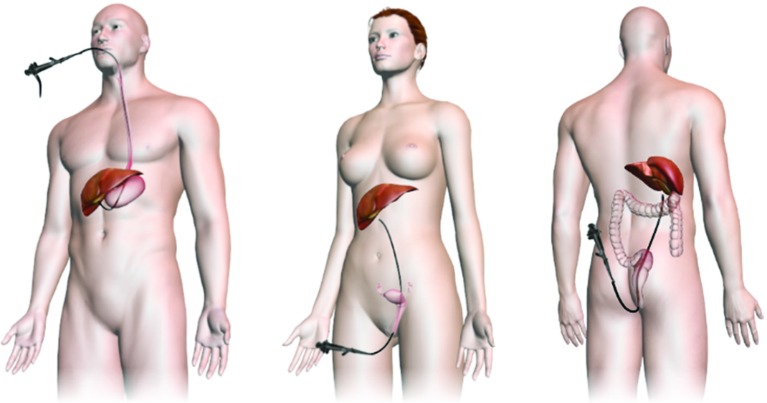
Trans-gastric (*left*), trans-vaginal (*middle*) and trans-rectal (*right*) NOTES approaches

Over the last 30 years, laparoscopic surgery has become the standard approach for many operative procedures. In order to push minimally invasive techniques further along the spectrum towards truly non-invasive surgery, surgeons have started using flexible endoscopy in procedures traditionally reserved for rigid instruments. By inserting a flexible endoscope via a natural orifice such as the oesophagus, vagina or anus (Fig. 1) and then navigating the endoscope through an internal incision in the relevant organ, surgeons can gain access to the abdominal cavity and are able to, for example, remove the gallbladder (cholecystectomy) or the appendix (appendectomy) leaving no external scars (incision/scar-less procedure). This emerging technique is known as natural orifice transluminal endoscopic surgery (NOTES). Since it eliminates external post-operative wounds, it is argued that NOTES may further reduce operation trauma, recovery time and clinical costs and improve overall cosmetic results, thereby pushing the boundaries of minimally invasive surgery (MIS) as we know it [1–4]. As with any new, potentially disruptive surgical technique, the benefits of NOTES are still to be fully realized, and there remains considerable dissent as to its true benefits and risks [5, 6]. The Natural Orifice Surgery Consortium for Assessment and Research (NOSCAR) established a list of potential barriers which need to be surpassed before NOTES can be incorporated into routine practice [7]. One of the key issues identified by specialists was the lack of efficient training programs available for clinicians and the extremely steep learning curve of NOTES procedures.

The novel use of a flexible endoscope in NOTES procedures differs substantially from both conventional endoscopy and laparoscopy. In addition to entering the abdomen through a natural orifice, the NOTES technique requires the surgeon to operate the endoscope and any associated instrumentation through a single access point, rather than the three or four ports common to a laparoscopic operation. Although many NOTES procedures are currently being performed in a hybrid fashion (i.e. with some trans-abdominal assistance), there is a significant loss of retraction and the in-line instrument approach through the instrument ports in the endoscope handpiece is unfamiliar to most surgeons. Another significant difference is the lack of a gastrointestinal lumen to support the endoscope. Thus, the distal end of the endoscope is manipulated in the open abdominal cavity using the incision (viscerotomy) site, internal organs and gravitational force to navigate and position the instrument. The middle section of the endoscope shaft can unpredictably roll and loop inside the abdomen. As a result of these differences in endoscope behaviour, the approach to regions of interest can also be very different in comparison with traditional endoscopic techniques. Taking all of the above into consideration, it is clear that performing NOTES procedures requires a new set of skills. Learning and practicing these skills demands a new set of training tasks supported by suitable simulator models. The most recent survey on education and training in NOTES [8] reviews 11 non-animal studies, 8 animal studies and 6 educational programs for NOTES. Several of them demonstrate construct validity. Most notable is the “ELITE” simulator—an ex vivo, full-scale replica of a female adult with various transluminal access points [9, 10]. The survey also states that minimal work has been carried out in the field of virtual reality (VR).

### VR simulation

Medical VR simulators provide a safe environment in which clinicians can repetitively practice without putting patients at risk. They have been expected to become an important part of surgical training since the early 1990s [11]. Recent reviews show that, although VR simulation is now successfully used in various surgical specialities, there is still enormous potential for further development [12–14]. A recent needs analysis for a NOTES VR simulator shows that there is indeed interest in such technology [15]. Whilst there are well-established, validated commercial VR simulators available for flexible endoscopy procedures (GI Mentor—www.simbionix.com; EndoVR—www.caehealthcare.com) such as lower/upper GI, endoscopic retrograde cholangiopancreatography (ERCP), endoscopic ultrasound (EUS) or flexible sigmoidoscopy (FS), simulating NOTES procedures requires more advanced dynamic modelling of the virtual endoscope so that it may operate in open abdominal cavities and interact with surrounding anatomy in a different manner.

In [16], Ahn and colleagues report the ongoing work on their Virtual Transluminal Endoscopic Surgery Trainer—VTEST$^{\mathrm{TM}}$. Their simulator aims to recreate a hybrid NOTES procedure using a rigid scope and a trans-vaginal approach. In [17], the same group describes their work on a prototype haptic device for flexible endoscope, but this is yet to be integrated into their VTEST$^{\mathrm{TM}}$ system and no results of its performance are presented. Therefore, to the best of our knowledge, there are currently no force-feedback-enabled, either commercial or experimental, VR simulators for NOTES procedures supporting a flexible endoscope.

In this paper, we provide a detailed technical description of our natural orifice virtual surgery (NOViSE) simulator, including its overall design, haptic interface, virtual flexible endoscope model, tool–tissue interactions, as well as implementation details. Next, we present results of the underlying endoscope model behaviour along with its computational performance. This is followed by the application of the NOViSE simulator to a hybrid trans-gastric cholecystectomy procedure (using a flexible endoscope) and a summary of previously reported initial validation results.

Our main contribution is in the development (software and hardware) and integration of a force-feedback-enabled virtual reality simulator for NOTES training supporting a flexible endoscope, together with the adaptation of the underlying Cosserat rod mathematical model to make it inextensible and incompressible like a real endoscope, as well as various performance optimization features to ensure reliable and stable real-time operation.

## Methods

NOViSE is a first prototype whose main focus is on teaching the endoscopic manipulation skills required for NOTES. The crucial components of such a simulator are the custom-built haptic device and the underlying mathematical model of the virtual flexible endoscope. As there is currently no established curriculum for NOTES, NOViSE was developed in close collaboration with experts who previously conducted a large number of NOTES animal and box model operations [8, 18], as well as a series of human NOTES trials.

Based on their experience, the procedure steps which are the most demanding in terms of endoscope manipulation were carefully identified. These are, namely navigation into the abdomen; clipping and cutting of a cystic duct and artery; and dissection of the gallbladder from the liver bed. The experts also identified the following NOTES-specific set of skills and challenges, which were to be the focus of the current NOViSE prototype, together with a set of relevant performance metrics (see relevant subsection below):

- Open space manipulations in the abdominal cavity
- Navigation with a lack of gastrointestinal lumen
- Single access point
- In-line instrument approach, i.e. instruments aligned with the main endoscope shaft
- Limited tissue retraction

Having identified the focus of the first NOViSE prototype, some trade-offs were recognized in order to streamline the development process and improve computational performance, without affecting the intended training outcomes, namely:

- Static, non-deformable models of oesophagus, stomach and intestines
- Static, pre-deformed model of a retracted liver
- Gallbladder and connective tissue modelled as mass–spring systems
- Empirical tuning of the (bio)-mechanical parameters of the endoscope and gallbladder

The following sections provide further details of the implementation and justification for the above trade-offs, including a summary of previously reported preliminary validation studies where their correctness and relevance were also assessed [19].

### Simulator set-up

**Fig. 2:**
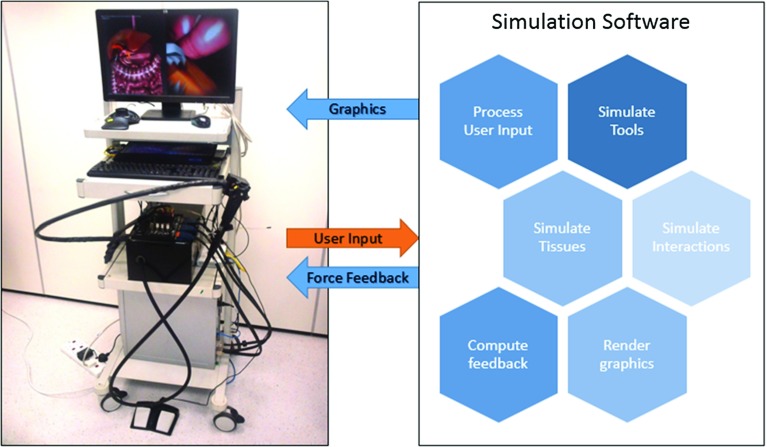
The complete NOViSE set-up (*left*). The overview of the simulation software sub-systems (*right*)

NOViSE (Fig. 2) consists of a real-time software simulation and a physical, force-feedback human–computer interface (haptic device). The software is written in Java, with performance critical sections implemented in C/C++. It can efficiently run, exceeding haptic interactive rates on a modern mid-range PC or laptop. The simulator display is divided into two parts. On the right, the user can see the endoscopic camera view. On the left, there is an external, optional “aerial view”, which can be turned on/off and freely manipulated.

**Fig. 3:**
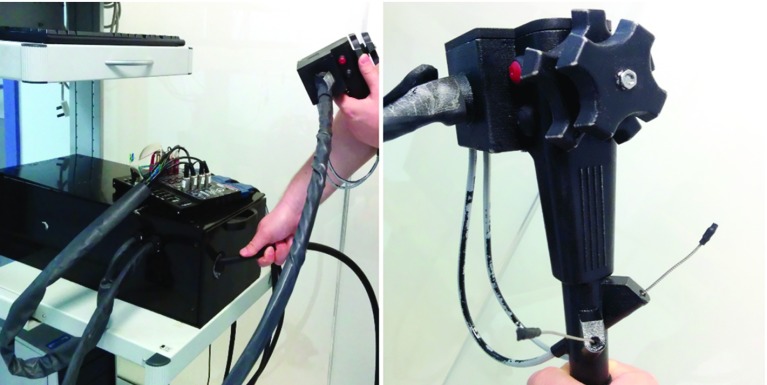
The haptic device connected to the data acquisition device (*left*). A close-up to the hand piece (*right*)

### Haptic device

Whilst there exist a variety of haptic interfaces for endoscopic simulation [20], we undertook a novel redesign with the aim of portability and affordability. Our haptic device (Fig. 3, left) comprises an enclosed black box of dimensions approximately 55 $\times$ 26 $\times$ 18 cm, into which passes a hose (1.5 m long, 15 mm diameter). The hose can be pushed or pulled through the opening (total travel 22 cm) and rotated freely. Inside the enclosure, the end of the hose is directly coupled to a 15:1 planetary gearbox and a servo motor delivering a combined total torque of $+$/− 2.55 Nm. This motor is mounted on a low-friction linear rail driven by an identical motor connected via a tensioned toothed drive belt and a 24 mm pulley resulting in $+$/− 14 N linear force output. Both linear (14 N) and rotational (2.55 Nm) force feedbacks significantly exceed the requirements for endoscopic NOTES procedures. These were measured by [17] and peak at 4.77 N for linear and only 0.03 Nm for rotational feedback.

At the proximal end of the hose, a 3D-printed plastic replica of a standard endoscopic handpiece is attached (Fig. 3, right). It consists of two force-feedback-enabled thumb wheels, two optically tracked thin wires representing the endoscopic tool wires and two push buttons. Additionally, a double foot pedal is placed on the floor and can be used to activate endoscopic instruments, e.g. diathermy. The thumbwheels are actuated using a common Bowden cable arrangement by two servo motors located in the main enclosure.

### Virtual flexible endoscope

The shaft of the virtual endoscope can be pushed, pulled and rotated through manipulating the haptic device. The tip of the virtual endoscope is steerable, with its bend controlled using two thumb wheels on the handpiece. The virtual scope is equipped with a light source, a camera and two working ports through which different instruments (actuators) may be inserted. Currently, operators can choose from four types of virtual actuators: grasper, clipper, scissors and diathermy tool. Their insertion/removal is controlled by two physical wires inserted in the two ports of the handpiece (Fig. 3, right).

A vital part of our simulator is the underlying mathematical model of the one-dimensional deformable body (elastic rod) responsible for the behaviour of the virtual flexible endoscope. Elastic rods are characterized by having large nonlinear deformations even if the local strains are small. This characteristic, as well as consideration of material twist and the fact that many rods practically do not stretch, makes their dynamic simulation challenging, especially in real-time. Physically based interactive approaches to elastic rods range from mass–spring models [21–23], rigid multi-body serial chains [24, 25], spline-based formulations [26], to Cosserat theory-based models [27–32]. For our flexible endoscope, we chose the CoRdE model by Spillmann and Teschner [29]: first, because it is based on Cosserat theory—a solid theoretical foundation considered as a final step in the formulation of a modern theory of elastic rods [33]; second, because CoRdE is a fast, dynamic and elegant solution with an explicit centreline representation, which facilitates the simulation of contacts. The explicit centreline also simplifies the overall implementation, internal friction calculations and rod visualization [29, 30]. CoRdE behaviour is independent of the underlying discretization. Additionally, this model was previously applied by our group to the simulation of catheters and guidewires in cardiovascular interventions with good results [34].

**Fig. 4:**
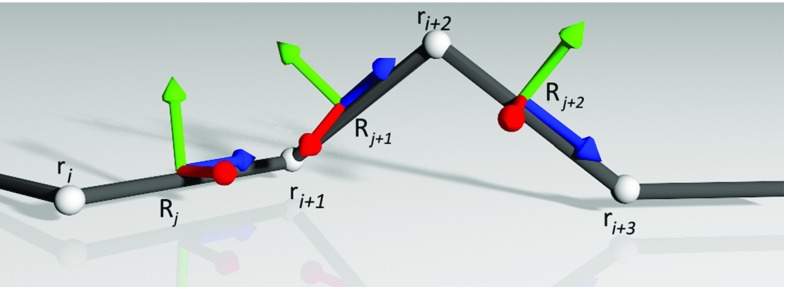
Material frames adapted to the Cosserat rod centreline

In CoRdE, a quaternion governing the material frames $\mathbf{R}_j \in {\mathbb {R}}^{3 \times 3}$ (cross-sectional orientation) is placed in between the neighbouring mass points $\mathbf{r}_i \in {\mathbb {R}}^{3}$ consisting of the centreline as shown in Fig. 4. By comparing the material frames, the bend and twist deformations can be quantified and the total elastic energy of the rod *V* can be derived. By minimizing *V*, the bending and twisting strain rates are coupled together in a unified manner. The twist deformation is balanced out by the bend deformation and vice versa, which results in the looping phenomenon. We refer the reader to the original publication for further implementation details [29].

In the original model, the penalty method was used to govern the stretch of the centreline. Due to this, a reduction in the rod’s stretch and compressibility may introduce additional stiffness to the system requiring a smaller time step. To address this, we modified the original model to make it inextensible and incompressible like a real endoscope. The penalty method was replaced by a chain of distance constraints. For accuracy and performance, the constraints Jacobians were arranged in a tri-diagonal banded system of equations. As this system contains only equality constraints, it is efficiently solved using Gaussian elimination with partial pivoting in a global manner (i.e. all distance constraints at once) as a first step of each solver iteration by a conventional linear algebra library (LAPACK, www.netlib.org). In the next step, the collision constraints including Coulombian friction approximation are applied locally. If needed, the whole process may be repeated multiple times to improve simulation accuracy.

Our modified CoRdE implementation enables real-time simulation at haptic interactive rates, providing an efficient, unified bending, twisting and collisions handling, as well as a very fast response to user manipulations and an easy parameterization of the mechanical properties of the rod. It allows for the physical properties of our virtual endoscope, such as mass, diameter, resistance to bending and twisting to be derived empirically, under the supervision of expert clinicians, to match the behaviour of a real flexible scope.

### Tool–tissue interactions

During the design stage, in collaboration with the NOTES experts, a series of tests were conducted a to identify which organs and/or anatomical structures are relevant to the training of endoscope manipulation skills. In a hybrid trans-gastric cholecystectomy, the endoscope is introduced via the oesophagus, travels inside the stomach and finally goes through the viscerotomy site (incision on the wall of the stomach) to the abdominal cavity. Once in the abdominal cavity, the endoscope and its actuators can collide and interact with the gallbladder, liver, fatty connective tissue between these two, as well as the small and large intestine and the pancreas.

Having identified the relevant anatomy, the experts compared two versions of the first phase of the procedure with and without deformation of oesophagus and stomach. Due to the hollow nature of these organs, their deformations were simulated using mass–spring models. The experts agreed that, although the deformations added to the visual plausibility in the auxiliary view, they were hardly noticeable in the endoscopic view alone and therefore were deemed irrelevant to the training of manipulation skills for NOTES.

**Fig. 5:**
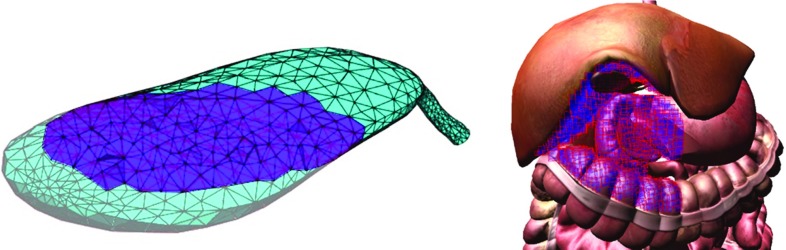
A cut through a tetrahedral mesh of the gallbladder (*left*). The abdomen tissues reachable by the endoscope wrapped in an AABB BVH tree (*right*)

After passing through the stomach and reaching the abdominal cavity, a rigid laparoscope is commonly used to retract the liver and the gallbladder. This involves deformation of the liver, connective tissue and gallbladder. Instead of attempting to model liver deformation in real time, which was deemed unnecessary given the focus of the current prototype, we “pre-modelled” the retracted liver using a 3D package as shown in Fig. 5, right.

Regarding the gallbladder, a 3D polygonal model was discretized into a tetrahedral mesh consisting of 1194 mass points, 3615 tetrahedrons and 5914 connecting springs using TetGen (www.tetgen.berlios.de). A mass–spring model (MSM) [35, 36] was implemented to simulate its deformation as it interacts with the endoscope and the actuators. Its body can be probed and grasped, and its cystic artery and duct can be clipped and cut. Additionally, the gallbladder can also be retracted in a conventional way using a rigid laparoscope (hybrid NOTES procedure). Its physical properties, such as spring stiffness and dampening, were visually and haptically tuned to approximate the behaviour of the real anatomy based on the judgement of senior clinicians interacting with the virtual anatomy through the haptic device, whilst achieving real-time performance. Although less accurate than continuum-based methods such as FEM, MSM can handle large deformations as well as topological modifications with relative ease. In spite of model behaviour being dependent on the underlying discretization and spring stiffness and damping parameters not directly correlating to measurable real-world values, MSM models are still often used to simulate deformable organs as they are fast and easy to implement.

The connective tissue between the gallbladder and the liver is also approximated by a MSM that can be dissected using the diathermy tool or scissors. Its physical properties were also fine-tuned by senior clinicians whilst interacting with the virtual anatomy through the haptic device. The deformations of other abdominal organs such as the intestines and pancreas were uniformly judged by the experts as rather irrelevant from the point of view of manipulations during trans-gastric cholecystectomy.

Collision detection between the endoscope or its actuators and surrounding tissues is based on a dynamic bounding volume hierarchy (BVH) of axis-aligned bounding boxes (AABB) as suggested in [37]. For performance reasons, only tissues which are in the region of interest of the operator, i.e. reachable by the endoscope (Fig. 5, right), are considered during collision detection. Gravity as a constant force in the supine position has been incorporated to both the gallbladder and the endoscope by considering acceleration due to gravity (*g* = 9.81 m/s$$^{2})$$ in the corresponding direction.

### Multi-threaded implementation

The simulator implementation targeted multi-core CPUs. It follows a task parallelism approach, in which the simulation tasks (rod physics computations, collision detection, deformable bodies simulation, scene graph transformations) are distributed across different threads. Different tasks can run at different rates relatively to the endoscope physics task. The collision detection task runs in parallel, one to one, a step behind, but the deformable body task runs approximately at 1/3 rate of the instrument physics. We use a form of double buffering to reduce the synchronization overhead and prevent concurrency issues such as visual flickering or physics instabilities. Our experiments show that task parallelism, in this case, results in approximately 50 % better performance compared with data parallelism, which spreads mass points and quaternions across the threads. This is caused by a smaller synchronization overhead, fewer CPU context switches and better cache utilization.

We also carried out tests using a GPU implementation (Intel Core2 2.66 GHz, NVidia GeForce GTX 560). The endoscope simulation poses a rather small problem in the context of massively parallel computations standards, and no performance gains were noted. In terms of soft body simulation, a reasonable x4–x5 speed-up was achieved for the gallbladder simulation.

However, when considering collision detection and interactions between the organs and the tools, the raw computational power of a GPU was largely mitigated by the overhead caused by memory transactions, kernel launches and CPU/GPU synchronization. Hence, it was decided to instead develop NOViSE targeting modern CPUs.

### Metrics

The NOViSE simulation software computes and collects a series of performance metrics (Table 1) which were derived during the analysis by the expert clinical collaborators of the particular tasks of hybrid trans-gastric cholecystectomy and endoscopic manipulation skills for NOTES. Once the procedure is complete, the simulator can present and process the metrics to generate a comma-separated (CSV) file with an adequate layout suitable to be imported into MS Excel or SPSS for further, more detailed analysis. In addition, for each completed task, an associated binary file containing the recorded motions of the haptic device and of the virtual endoscope is generated. From these, several supplementary metrics can be extracted or stored performances played back for further study.

## Results

In this section, we present brief qualitative and quantitative results of our elastic rod implementation in terms of its behaviour and performance. Next, we describe the application of the above methods in NOViSE to simulate a hybrid trans-gastric cholecystectomy procedure. Finally, we summarize the results of an initial face and content validity study.

**Table 1:** Metrics collected during the simulation

For all tasks
Task completion time
Path length of the tip traversed during the task
Maximum and average forces applied to tissues by endoscope
Maximum and average velocities and accelerations of the shaft
Maximum and average haptic force feedback
For clipping and cutting (sub)-tasks
Clipping/cutting distance from the indicated point
Clipping/cutting angle between the tool and the surface
Number of clippings /cuttings
Degree of instrument protrusion during the operation
For gallbladder dissection task
Number of diathermy activations
Total time diathermy activation time
Time diathermy activated on target/non-target tissues
Percentage of time burning non-target tissue

### Rod inextensibility and incompressibility

**Fig. 6:**
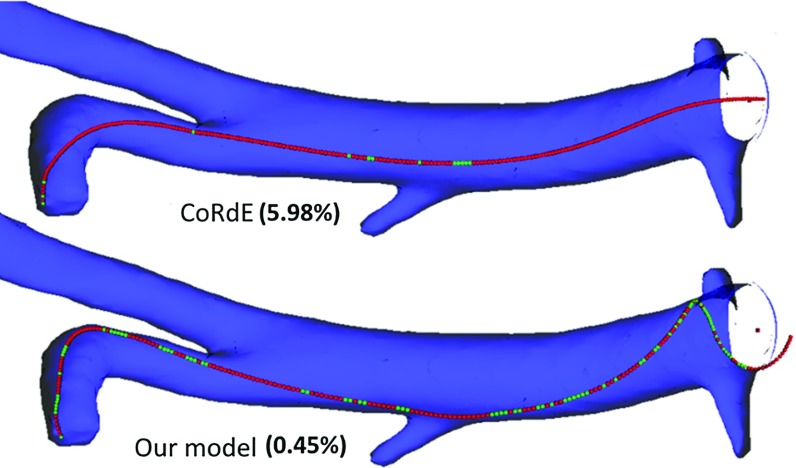
Rod compression test by insertion into a virtual vascular model. At the *top*: the original CoRdE model. At the *bottom*: our inextensible and incompressible modification. The percentage of compression is given in *brackets*. The *green spheres* represent colliding mass points and the *red ones* non-colliding

In order to demonstrate the importance of inextensibility and incompressibility, a rod consisting of 256 (e.g. 255 mm long) points was gradually inserted into a random rigid anatomy model in a way to aggravate its compression. Our modified rod compressed by less than 0.5 %. In contrast, the CoRdE model with highest possible (but stable) stretching Young’s modulus $$E_s $$ compressed by nearly 6 %. Such a compression is not only clearly noticeable, as evident in Fig. 6, but also results in a very different bend and twist deformation. The computational time required by our incompressible rod (0.86 ms per iteration, including collisions) was only 6 % longer than that of the original CoRdE model (0.81 ms).

### Computational performance

**Fig. 7:**
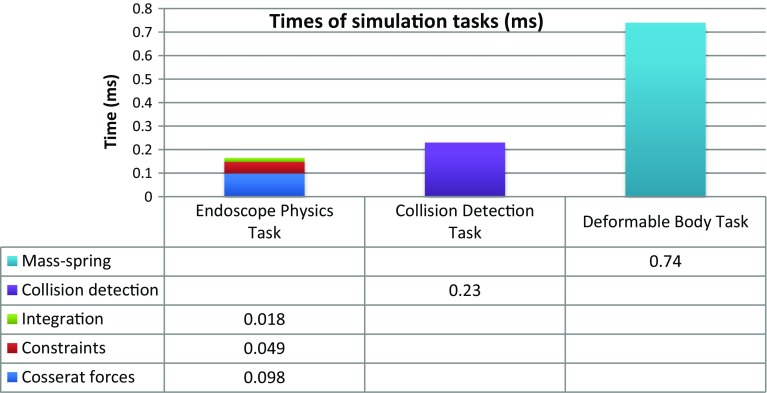
Computational times of different components on Asus N55s Laptop

The entire computational performance during the procedure was tested on an Asus N55s laptop (Win7 $\times$64, Intel Core i7 2.2 GHz, 8GB RAM, NVidia GeForce GT 555M). The total computational time of the virtual endoscope (Cosserat forces, constraints, integration) consisting of 100 mass points was below 0.2 ms. The collision detection runs in sync on a separate thread and slightly slows down the rod physics (0.23 ms). The mass–spring model for the deformable gallbladder was the slowest part of the simulation requiring nearly 0.75 ms per update. The display was updated at a standard 60 frames per second. In Fig. 7, we present the average computational times for each particular simulation (sub)-task: endoscope physics, collision detection and deformable body simulation.

Due to different hardware platforms and test cases used, the computational performance of our rod cannot be directly compared to other Cosserat rod implementations found in the literature. However, approximate comparisons indicate that the performance of our approach is similar to the original stretchable CoRdE [29]. Yet, considering the inextensibility, our rod was roughly $\times$4, $\times$38, $\times$24 time faster than the approaches presented in [30, 38] and [39], respectively. However, these models have other advantages, for instance, the elimination of the penalty method in parallel constraints, improved stability or better contact handling. Table 2 summarizes these results.

**Table 2:** Comparison of computational times in respect to other models

Model	PC CPU	Time stated in paper (ms/pts)	Time (ms/100 pts)	Speed-up
Our inextensible model	Core2 2.66 GHz	0.147/100	0.147	$\times$1.00
Original CoRdE [29]	Xeon 3.80 GHz	0.131/100	0.131	$\times$0.89
Inextensible CoRdE [38]	Core2 3.00 GHz	2.26/40	5.65*	$\times$38.4*
Discrete elastic rods [30]	Core2 2.66 GHz	0.34/75−0.42/67	0.45*−0.67*	$\times$3.06$-\times$4.56*
Position based elastic rods [39]	N/A	1.06/30	3.53*	$\times$24.0*

* Approximated times

### Hybrid trans-gastric cholecystectomy

**Fig. 8:**
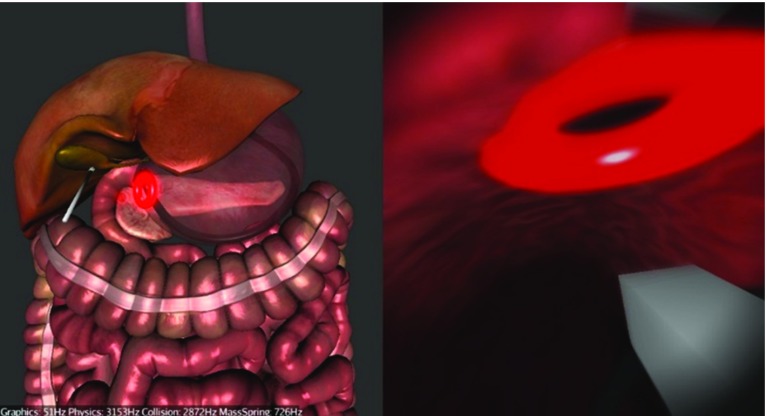
Task 1—Navigation from the stomach into the abdomen via the incision (viscerotomy) site (*glowing red ring*)

**Fig. 9:**
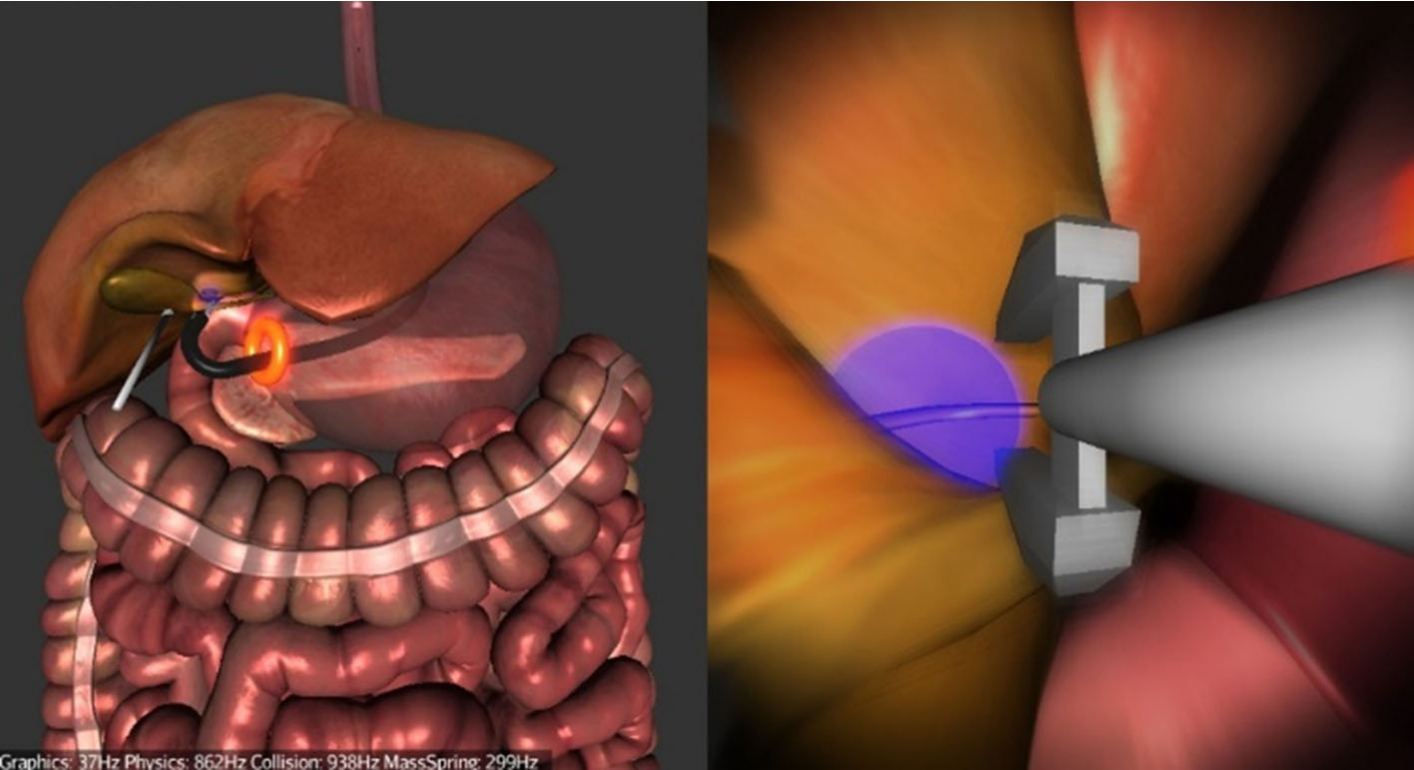
Task 2—Clipping the cystic artery

**Fig. 10:**
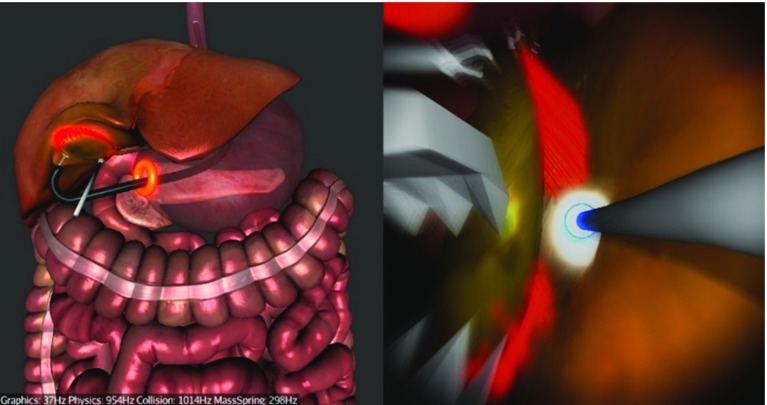
Task 3—Gallbladder dissection using diathermy. The *red line* segments represent the connective tissue. An auxiliary laparoscope retracting the gallbladder is visible

We chose to simulate a hybrid trans-gastric cholecystectomy since laparoscopic cholecystectomy (gallbladder removal) is one of the most prevalent surgical interventions. It was also amongst the first NOTES procedures, and it is currently one way in which NOTES is performed in clinical practice. It is a hybrid operation as laparoscopic assistance for visualization and retraction is coupled with a flexible endoscope. The simulation starts with the endoscope partially inserted into the oesophagus. It is divided into three main tasks: navigation via the stomach to the abdomen (Fig. 8), clipping and cutting of the Calot’s triangle (Fig. 9) and gallbladder dissection using the diathermy tool and the grasper (Fig. 10). The operator is guided by glowing markers indicating an optimal path, an incision (viscerotomy) site, clipping points/angles and connective tissue.

During the first task (Fig. 8), the operator needs to find the viscerotomy site located on a side of the stomach and navigate the scope through it into the abdominal cavity. S/he is not required to pierce the stomach as the incision is already present and represented by a glowing red ring. However, navigating through the ring is not trivial as it requires a combination of bimanual motions of the endoscope shaft and handpiece controls.

After entering the abdomen, the operator can proceed to the second task—clipping and cutting of the cystic artery and duct (Fig. 9). The operator needs to locate an anatomical region called Calot’s triangle and start by clipping the cystic artery first. The optimal clipping point is indicated by a blue marker where the operator must insert a clipping tool, positioning its jaws as close to the blue marker on the artery as possible, whilst maintaining a right angle between the jaws and the artery. Next, s/he needs to place another clip on the artery and cut between the clips using scissors. This clip-and-cut process is repeated on the cystic duct. The key to completing this task efficiently is to correctly navigate and position the tool right from the start so that all the clipping and cutting can be done without having to manipulate the endoscope. This way, all the six points of interest should be within reach by only adjusting the tip of the scope and inserting/removing the actuators.

Having clipped and cut the cystic duct and the artery, the operator can progress to dissect the connective tissue between the gallbladder and the liver bed using the diathermy tool (Fig. 10). The connective tissue is represented by red glowing line segments that are burnt by the operator activating the diathermy tool close to them. Activation of the diathermy needs to be precise and accurate in order to burn as little of other non-target tissues as possible.

**Fig. 11:**
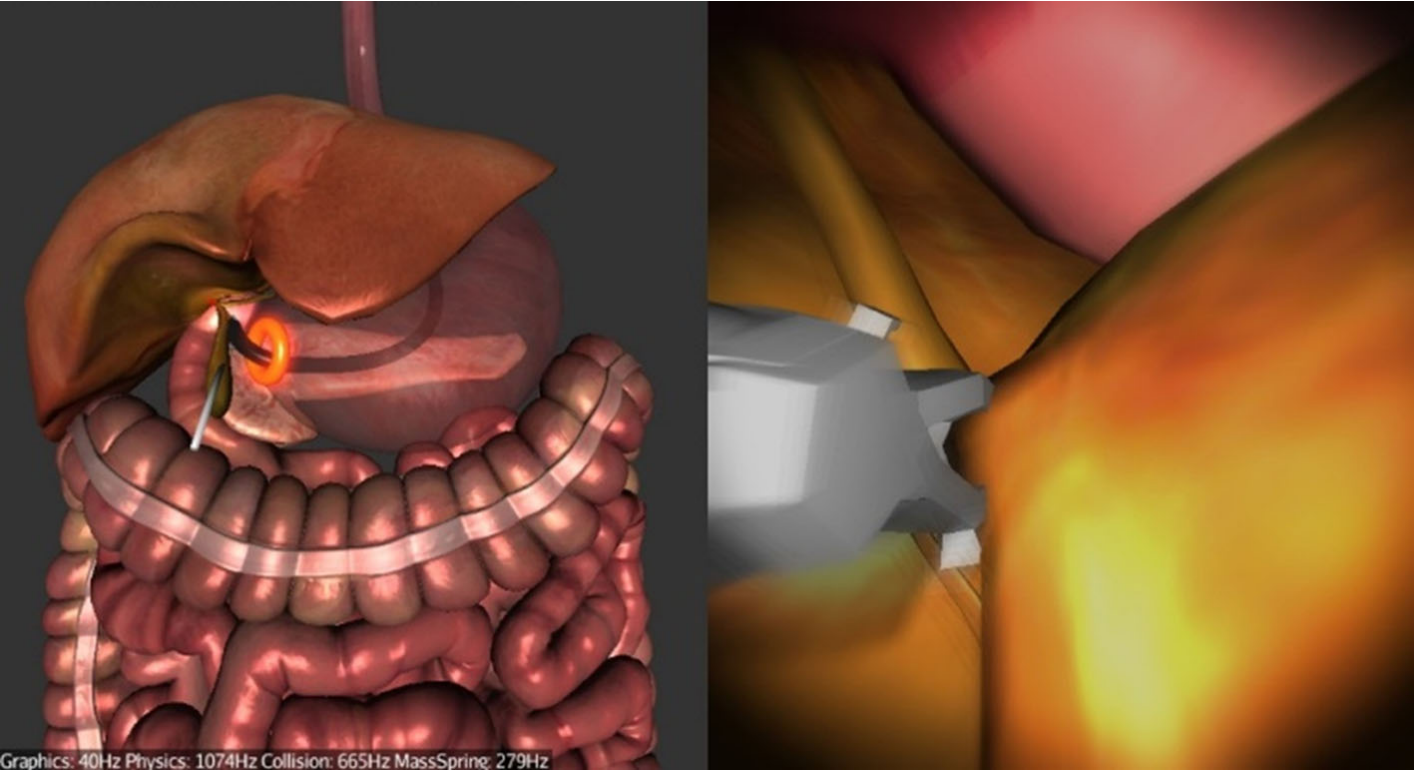
Completing the procedure by pulling out the gallbladder using the grasper

As mentioned above, the simulated procedure is hybrid, which means that there is still one laparoscopic instrument deployed in the conventional way used to retract the gallbladder in order to get a better exposure of the connective tissue. This retraction is controlled using the keyboard by the assistant given the direct command to prevent bias. After removing the connective tissue, the operator can use the actuator grasper to hold the gallbladder and pull it out through the stomach (Fig. 11). At this point, the procedure is completed. The operator is not required to close the viscerotomy site.

In between certain tasks, the screen occasionally fades out and the simulation is paused. When this happens, the operator is asked to adjust the insertion of the hose so that s/he will have enough insertion/retraction available to complete each task without reaching the limit of the haptic device.

### Validation

An initial verification of the clinical realism and accuracy of the instruments was previously carried out by obtaining subjective feedback (face and content validity) through a questionnaire from 14 surgeons in different specialities [19]. Four of them were qualified as NOTES experts who have performed 10 or more human or animal model NOTES procedures independently. A summary of the validation results is presented below.

**Fig. 12:**
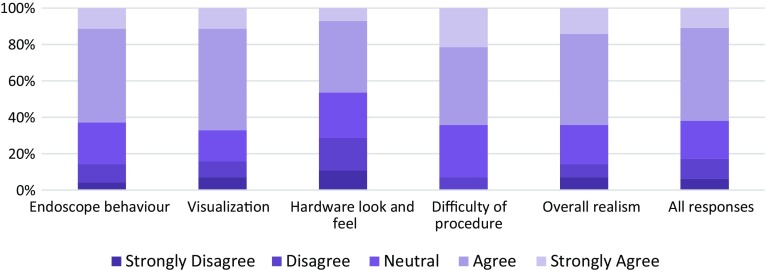
Face validity results—participants’ responses [19]

NOViSE showed good overall face validity (Fig. 12). In the questionnaire, 63 % of responses to statements regarding the realism of the virtual endoscope and 67 % of responses to statements regarding the visual realism of the endoscopic camera were “agree” or “strongly agree”. The participants were most critical of the overall look and feel of the hardware. 64 % of participants stated (i.e. agreed or strongly agreed) that the simulator and the difficulty of the simulated procedure were realistic.

**Fig. 13:**
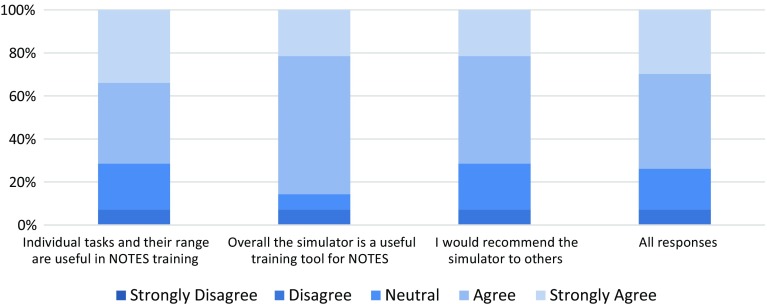
Content validity results—participants’ responses [19]

NOViSE showed also good content validity (Fig. 13). In the questionnaire, 74 % of responses to the statements assessing the usefulness and range of the individual tasks for training were “agree” or “strongly agree”. Majority (86 %) of the participants stated (i.e. agreed or strongly agreed) that NOViSE is a useful training tool for NOTES and 71 % that they would recommend it to others.

**Fig. 14:**
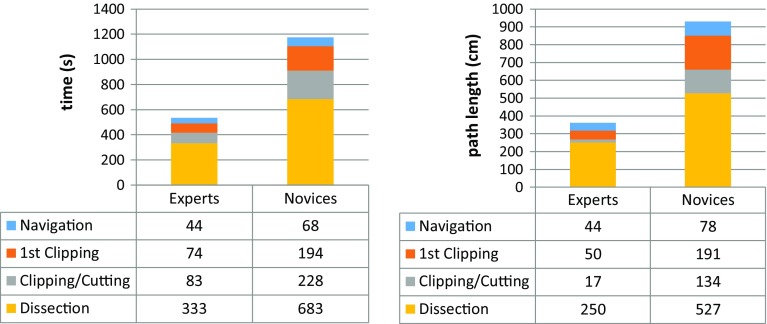
Construct validity results—task completion times (*left*) and economy of movement (*right*) [19]

Regarding construct validity, experts were faster and had better economy of movement (i.e. used a shorter endoscopic path length) than novices in all but the first task. Due to the non-normal distribution of data, a nonparametric Mann–Whitney *U* test was used to compare the performance between groups. The statistical significance was set at *p* < 0.05, and it was demonstrated for the following tasks and metrics: time and economy of movement (EoM) from exiting stomach to application of first clip (74 vs. 194 s *p* = 0.04, 50 vs. 191 cm, *p* = 0.01), time and EoM from application of first clip to start of dissection (83 vs. 228 s *p* = 0.04, 17 vs. 134 cm, *p* = 0.04), time from application of last clip to completed dissection of gallbladder from liver bed (333 vs. 683 s *p* = 0.02, 250 vs. 527 cm, *p* = 0.04). The remaining metrics did not demonstrate statistically significant differences between experts and novices (Fig. 14).

## Limitations and future work

A main limitation of NOViSE is that it currently simulates only trans-gastric hybrid cholecystectomy. Supporting a wider range of procedures (e.g. appendectomy) and approaches (trans-vaginal, possibly trans-rectal), as well advanced endoscopic interventions is an important next step in its development. Given that NOViSE is a first prototype, it exhibits some hardware and software limitations. Replacing the physical endoscope hose for a lighter and softer one is required, together with modifications to the design of the handpiece.

During the virtual procedure, there is lack of fat tissue surrounding the Calot’s triangle. The steps of creation and closure of the viscerotomy site are omitted. Adding these features and tasks could recreate a more comprehensive NOTES experience. The soft body model used for simulating the gallbladder is relatively simple, whilst all the other organs are static and do not deform. It would be interesting to investigate further whether using more advanced deformation models (such as FEM), not just for the gallbladder, but possibly also for the oesophagus, stomach and liver would make a perceptible difference to the user experience in the context of the whole procedure. Manipulating the patient’s position on the operating table, as it is done in flexible endoscopy, and adjusting the resulting gravitational force could be another potential further refinement of the simulator.

Although initial tests in the current prototype showed no benefits from using a massively parallel GPU implementation, considering the rapid progress in the field and the possible need for further and more advanced organ defamation modelling, this may need to be re-evaluated. More specifically, a unified physics approach [40] might be worth exploring.

Additional validation studies are foreseen after implementing the suggested modifications to both hardware and software components of NOViSE. We would like to evaluate the feasibility of our platform for the simulation of advanced endoscopic procedures such as endoscopic mucosal resection (EMR), endoscopic submucosal dissection (ESD) and per-oral endoscopic myotomy (POEM).

## Conclusions

In this paper, we have presented a prototype virtual reality force-feedback-enabled simulator for NOTES, which supports a trans-gastric hybrid cholecystectomy procedure using a flexible endoscope. At this stage of development, the focus has been on teaching of specific endoscopic manipulations required for NOTES, yet NOViSE has established promising foundations for further development. The operator interacts with the virtual endoscope via a custom-built haptic device. The behaviour of the virtual flexible endoscope is based on the Cosserat theory, allowing for realistic recreation of bending and twisting of the virtual endoscope, as well as guaranteeing a fast response to user manipulations and incorporating gravity as a constant force in the supine position. The efficient, multi-threaded implementation enables the simulation to run efficiently on an off-the-shelf PC or laptop at haptic interactive rates.

In a preliminary validation study [19], NOViSE has shown good overall face and content validity, with improvements suggested to the feel of the haptic device and design of the handpiece. Participants agreed that NOViSE is sufficiently realistic, that it can be a useful training tool for NOTES and that they would recommend it to others. NOViSE also demonstrated early signs of construct validity. Experts were faster and had better economy of movements than novices in 3 out of 4 tasks than novices.

These initial results indicate that NOViSE can recreate a trans-gastric hybrid cholecystectomy procedure, potentially contributing to surgical training and to improving the educational experience for NOTES, without putting patients at risk, raising ethical issues or requiring expensive animal or cadaveric facilities. Moreover, considering that NOTES is still an experimental technique without an established curriculum, NOViSE offers the possibility to facilitate its development through VR simulation, for instance, in pre-operative planning or prototyping of new surgical devices. NOViSE could also potentially support the wider adoption of NOTES and advanced endoscopy procedures by keeping practitioners up to date with these novel minimally invasive surgery techniques.

## Electronic supplementary material

Below is the link to the electronic supplementary material.

Supplementary material 1 (mov 65725 KB)
